# Updated safety results from phase 3 lecanemab study in early Alzheimer’s disease

**DOI:** 10.1186/s13195-024-01441-8

**Published:** 2024-05-10

**Authors:** Lawrence S. Honig, Marwan N. Sabbagh, Christopher H. van Dyck, Reisa A. Sperling, Steven Hersch, Andre Matta, Luigi Giorgi, Michelle Gee, Michio Kanekiyo, David Li, Derk Purcell, Shobha Dhadda, Michael Irizarry, Lynn Kramer

**Affiliations:** 1 Columbia University Irving Medical Center, NYS Center of Excellence for Alzheimer’s Disease, Taub Institute for Research on Alzheimer’s Disease and the Aging Brain, Gertrude H. Sergievsky Center (PH19), & Department of Neurology, Columbia University Vagelos College of Physicians & Surgeons, 630 West 168th Street (P&S UNIT 16), New York, NY 10032-3795 USA; 2 Barrow Neurological Institute, Phoenix, AZ 85013 USA; 3 Yale School of Medicine, New Haven, CT USA; 4 Brigham and Women’s Hospital, Massachusetts General Hospital, Harvard Medical School, Boston, MA USA; 5 Eisai Inc., Nutley, NJ USA; 6 Eisai Co., Ltd, Hatfield, UK; 7 Clario, Philadelphia, PA USA

**Keywords:** Alzheimer’s disease, Lecanemab, Safety, ARIA

## Abstract

**Background:**

Alzheimer disease (AD) is a major health problem of aging, with tremendous burden on healthcare systems, patients, and families globally. Lecanemab, an FDA-approved amyloid beta (Aβ)-directed antibody indicated for the treatment of early AD, binds with high affinity to soluble Aβ protofibrils, which have been shown to be more toxic to neurons than monomers or insoluble fibrils. Lecanemab has been shown to be well tolerated in multiple clinical trials, although risks include an increased rate of amyloid-related imaging abnormalities (ARIA) and infusion reactions relative to placebo.

**Methods:**

Clarity AD was an 18-month treatment (Core study), multicenter, double-blind, placebo-controlled, parallel-group study with open-label extension (OLE) in participants with early AD. Eligible participants were randomized 1:1 across 2 treatment groups (placebo and lecanemab 10 mg/kg biweekly). Safety evaluations included monitoring of vital signs, physical examinations, adverse events, clinical laboratory parameters, and 12-lead electrocardiograms. ARIA occurrence was monitored throughout the study by magnetic resonance imaging, read both locally and centrally.

**Results:**

Overall, 1795 participants from Core and 1612 participants with at least one dose of lecanemab (Core + OLE) were included. Lecanemab was generally well-tolerated in Clarity AD, with no deaths related to lecanemab in the Core study. There were 9 deaths during the OLE, with 4 deemed possibly related to study treatment. Of the 24 deaths in Core + OLE, 3 were due to intracerebral hemorrhage (ICH): 1 placebo in the Core due to ICH, and 2 lecanemab in OLE with concurrent ICH (1 on tissue plasminogen activator and 1 on anticoagulant therapy). In the Core + OLE, the most common adverse events in the lecanemab group (> 10%) were infusion-related reactions (24.5%), ARIA with hemosiderin deposits (ARIA-H) microhemorrhages (16.0%), COVID-19 (14.7%), ARIA with edema (ARIA-E; 13.6%), and headache (10.3%). ARIA-E and ARIA-H were largely radiographically mild-to-moderate. ARIA-E generally occurred within 3–6 months of treatment, was more common in ApoE e4 carriers (16.8%) and most common in ApoE ε4 homozygous participants (34.5%).

**Conclusions:**

Lecanemab was generally well-tolerated, with the most common adverse events being infusion-related reactions, ARIA-H, ARIA-E. Clinicians, participants, and caregivers should understand the incidence, monitoring, and management of these events for optimal patient care.

**Trial registration:**

ClinicalTrials.gov numbers: Clarity AD NCT03887455)

**Supplementary Information:**

The online version contains supplementary material available at 10.1186/s13195-024-01441-8.

## Background

Alzheimer disease (AD) is a major health problem in older individuals, with tremendous burden on healthcare systems, patients, and families globally [1–5]. Amyloid beta (Aβ) has been identified as having an important role in the pathogenesis of AD based on the evidence of it likely playing an important role in the development and progression of the disease [6]. Disease modifying therapies are now approved that can improve the lives of those with early AD and slow progression of the disease [7–10]. 

Lecanemab, an FDA-approved amyloid beta-directed antibody indicated for the treatment of early AD (i.e., mild cognitive impairment or mild dementia stage of disease), binds with high affinity to soluble Aβ protofibrils, which have been shown to be more toxic to neurons than monomers or insoluble fibrils [11–15]. A large, 18-month phase 2, proof-of-concept, dose-finding study using a Bayesian adaptive design was conducted in 856 participants with mild cognitive impairment (MCI) due to AD and mild AD dementia (collectively defined as early AD) [8]. Results from this study showed evidence of clinical efficacy as well as an increase in incidence of amyloid-related imaging abnormalities (ARIA) versus placebo. [7,16,17]

Based on the results from the dose-ranging phase 2 study, a randomized, double-blind, placebo-controlled Core phase 3 study (Clarity AD) with an open-label extension (OLE) was initiated to confirm the efficacy and safety of lecanemab in participants with early Alzheimer’s disease [7]. In Clarity AD, change from baseline for lecanemab in the primary outcome of Clinical Dementia Rating-Sum-of-Boxes (CDR-SB) scores was less than for placebo at 18 months, with all key secondary clinical outcomes supporting the primary outcome. Lecanemab has been shown to be well tolerated in multiple clinical trials [7,8,17] but was associated with an increase in amyloid-related imaging abnormalities (ARIA) and infusion reactions relative to placebo. ARIA-E (ARIA with edema) and ARIA-H (ARIA with hemosiderin deposition: microhemorrhage and superficial siderosis) both appear to relate to the presence of cerebral amyloid angiopathy (CAA) and ApoE e4, can occur spontaneously in AD, and occur at increased rates relative to the background rates in the setting of amyloid-modifying therapeutic approaches [18]. The most common adverse events (> 10%) in the lecanemab group in Clarity AD were infusion reactions (lecanemab:26.4%; placebo:7.4%), ARIA-H (combined superficial siderosis and cerebral microhemorrhages; lecanemab:16.9%; placebo:8.9%), ARIA-E (lecanemab:12.6%; placebo:1.7%), headache (lecanemab:11.1%; placebo:8.1%), and fall (lecanemab:10.4%; placebo:9.6%).

Herein, we present safety observations along with more detailed safety data from the double-blind Core phase and the OLE of the phase 3 Clarity AD in early Alzheimer’s disease. The focus of this paper will be on safety results for lecanemab treatment from the combined Core + OLE, including serious adverse events (SAEs), deaths during the study, and updated ARIA data to complement previously published safety data from the phase 2^8,17^ and phase 3 Core [7] studies.

## Methods

### Participants

The methods and primary results for Clarity AD have been previously published [7]. Briefly, eligibility criteria included age 50 to 90 years old, MCI due to AD, or mild AD dementia based on National Institute of Aging–Alzheimer’s Association (NIA-AA) criteria, [19,20] with amyloid pathology confirmed by amyloid positron emission tomography (PET) or cerebrospinal fluid (CSF). All participants were required to have objective impairment in episodic memory as indicated by at least 1 standard deviation below the age-adjusted mean in the Wechsler Memory Scale IV-Logical Memory (subscale) II. Enrollment criteria required that screening MRI showed no more than 4 microhemorrhages and no extensive white matter pathology. Subjects were excluded if they had a bleeding disorder that was not under adequate control (including a platelet count < 50,000, or international normalized ratio > 1.5 for subjects who were not on anticoagulant treatment, e.g., warfarin). Subjects who were on anticoagulant therapy had to have their anticoagulant status optimized and be on a stable dose for 4 weeks before screening for the study. Participants from either treatment group who completed the Core were eligible to receive lecanemab in the OLE. The treatment assignment in the double-blind phase was not disclosed to the patients or study teams at the initiation of the OLE phase, nor until all patients in OLE phase had completed at least 6 months of OLE. Additional entry criteria are summarized in van Dyck 2023 [7]. 

### Trial design and oversight

Clarity AD was an 18-month treatment (Core study), multicenter, double-blind, placebo-controlled, parallel-group study with OLE in participants with early AD. Eligible participants were randomized across 2 treatment groups (placebo and lecanemab 10 mg/kg biweekly) according to a fixed 1:1 schedule. At the end of the Core study, individuals from either Core treatment group who participated in the OLE received lecanemab 10 mg/kg biweekly.

The studies were conducted in accordance with International Conference on Harmonisation guidelines and ethical principles of the Declaration of Helsinki. All participants and their study partners provided written informed consent.

### Safety assessments

Safety was monitored throughout the Core study in a blinded manner by the sponsor and in an unblinded manner by an independent data safety monitoring committee. Safety evaluations included monitoring of vital signs, physical examinations, adverse events, clinical laboratory parameters, and 12-lead electrocardiograms. ARIA occurrence was monitored throughout the study. Investigators responsible for medical management of participants were different from those involved in clinical assessments. ARIA occurrences were monitored throughout the Core trial by local and central reading of magnetic resonance imaging (MRI) performed at weeks 9, 13, 27, 53, and 79, as well as, for terminating subjects, a 3 month follow up visit for safety monitoring. An independent medical monitoring team was utilized to manage ARIA, infusion-related reactions and hypersensitivity reactions and they were firewalled from the clinical team managing the study. ARIA was evaluated for radiographic severity by the central reader (ARIA-E definitions are provided in Table S1).

In the OLE, safety was monitored at each infusion/visit and all participants followed a similar safety MRI schedule as in the Core (at 11 weeks, 15 weeks, and 6 months after the start of the OLE). All participants underwent non-contrast brain MRI during the screening period and randomization phase, at the visits specified in the clinical protocol. Scans were collected from 1.5T or 3T MRI scanners, using a standardized MRI protocol including a sagittal 3D T1-weighted sequence (General Electric 3D IR-prep Fast SPGR, Philips 3D TFE, Siemens 3D MPRAGE), with a 1.25 × 1.25 × 1.2 mm image resolution, and 2D axial T2- fluid attenuation inversion recovery (FLAIR), T2* Gradient Echo, T2 Turbo Spin Echo and Diffusion-Weighted scans (5 mm slices, 0.5 mm interslice gap, 240 mm Field of View, 256 × 256 matrix, or in some cases 128 × 128). All scanners were pre-qualified by Clario (Philadelphia PA), and all MRI scans underwent a thorough ongoing Quality Control (QC). T2-FLAIR was used for ARIA-E monitoring, while T2* was used for ARIA-H assessment. The 3DT1 sequence was mainly used for quantitative assessment of brain volume changes. The remainder of the protocol helped characterize the presence of any focal lesions including, but not limited to, evidence for microhemorrhages and intracerebral hemorrhages (ICH), superficial siderosis, ischemic and hemorrhagic stroke, subdural hematoma, neoplasm, arteriovenous malformation, lacunar infarcts and white matter abnormalities.

### Statistical analysis

Treatment duration of the double-blind Core study was 18 months with a 3 month follow up. The OLE study is still ongoing with an expected treatment duration of 4 years. Safety was evaluated in the safety analysis set, which was the group of participants who received at least one dose of study drug. OLE analyses include the group of participants that received at least one dose of lecanemab in the Core study and/or extension study. ARIA-E and ARIA-H data were summarized according to observed events and modelled via Kaplan-Meier graphs.

Additional details on the design and analysis methods are provided in van Dyck 2023 [7]. 

## Results

### Participants

Data presented from the Core includes 1795 participants from Clarity AD double blind, 897 randomized to placebo and 898 randomized to lecanemab. Data from the Core + OLE includes 1612 participants with at least one dose of lecanemab, 898 participants randomized to lecanemab in the Core and 714 participants who received placebo in Core and then converted to lecanemab in OLE. Of the 1612 lecanemab-treated participants, 1321 had exposure of greater than or equal to 6 months, 1007 participants had exposure of greater than or equal to 12 months, 505 participants had exposure greater than or equal to 24 months and 47 participants had exposure of greater than or equal to 36 months in this data cut off (as of December 1, 2022).

Baseline characteristics for those in the Core + OLE were generally similar to the characteristics in both treatment groups of the Core study (Table 1). The Core + OLE population had a mean age of 71.5 years, were 52.4% female, and 76.2% Caucasian. Overall, 69.3% of participants were ApoE ε4 carriers (53.8% heterozygotes; 15.4% homozygotes). In the Core + OLE, baseline antithrombotic use was 36.5% and the most common antithrombotic medication was acetylsalicylic acid (27.5% overall and 75.2% of those individuals with concomitant antithrombotic medication use). Overall, the Clarity AD study was conducted in patients with broad range of comorbidities and concomitant medications, from a diverse racial and ethnic background, and from clinical trial practice settings similar those for the general population.

**Table 1:** Characteristics of participants at baseline in the intent-to-treat population

	Core		Core + OLE
	Placebo (*N* = 897)	Lecanemab 10 mg/kg biweekly (*N* = 898)	Lecanemab 10 mg/kg biweekly (*N* = 1612)
Age, mean (standard deviation), years	71.1 (7.8)	71.4 (7.9)	71.5 (7.8)
Female, n (%)	476 (53.1)	462 (51.4)	844 (52.4)
Male, n (%)	421 (46.9)	436 (48.6)	768 (47.6)
Race, n (%)			
Caucasian	696 (77.6)	685 (76.3)	1228 (76.2)
Black	25 (2.8)	22 (2.4)	40 (2.5)
Asian	150 (16.7)	153 (17.0)	282 (17.5)
Other	26 (2.8)	38 (4.2)	62 (3.8)
Ethnicity, n (%)			
Hispanic	114 (12.7)	117 (13.0)	190 (11.8)
Years since diagnosis, mean (standard deviation), years	1.3 (1.5)	1.4 (1.5)	1.4 (1.5)
Years since onset of symptoms, mean (standard deviation), years	4.2 (2.5)	4.1 (2.4)	4.2 (2.4)
Mild dementia due to Alzheimer’s disease	342 (38.1)	346 (38.5)	607 (37.7)
Mild cognitive impairment	555 (61.9)	552 (61.5)	1005 (62.3)
ApoE e4 Status			
Noncarrier	286 (31.9)	278 (31.0)	495 (30.7)
Carrier	611 (68.1)	620 (69.0)	1117 (69.3)
Heterozygous	478 (53.3)	479 (53.3)	867 (53.8)
Homozygous	133 (14.8)	141 (15.7)	249 (15.4)
Ongoing treatment with AChEIs and/or memantine	477 (53.2)	466 (51.9)	876 (54.3)
Baseline microhemorrhage, n (%)	159 (17.7%)	141 (15.7%)	274 (17.0%)

CDR = Clinical Dementia Rating; ApoE e4 = apolipoprotein E – e4; AChEIs = acetylcholinesterase inhibitors; ADCOMS = the Alzheimer’s Disease Composite Score; ADAS-Cog14 = Alzheimer’s disease Assessment Scale-Cognitive Subscale; CDR-SB = Clinical Dementia Rating-Sum-of-Boxes; MMSE = Mini Mental State Examination; PET SUVr = positron emission tomography standard uptake value ratio. *At OLE baseline, we have 3 participants with CDR Global = 0, 51 participants = 2, and 7 participants = 3

### General safety update

General safety outcomes for Core + OLE can be found in Table 2, which also include those from the previously published Core study for comparison [7]. In the Core + OLE, deaths occurred in 1.0% and SAEs were experienced by 15% of participants. The occurrences of deaths or SAEs were similar regardless of ApoE ε4 genotype (Table S2). In the Core, there were 7 deaths on placebo (0.8%) and 6 on lecanemab (0.7%), and none were considered related to study drug. Two additional deaths (1 placebo and 1 lecanemab) occurred 30 days after last study treatment administration in the Core. In the OLE treatment period, there were 9 additional deaths as of data cut off for this manuscript, with 4 deemed possibly related to study treatment. Of the 24 deaths in placebo or lecanemab treatment groups across the Core + OLE, 3 were due to intracerebral hemorrhage: 1 placebo in the Core due to intracerebral hemorrhage (ICH), and 2 lecanemab in OLE with concurrent ICH (1 after tissue plasminogen activator [tPA] and 1 on anticoagulant therapy). Exposure-adjusted rates of death for lecanemab in the Core + OLE was 0.0069 per participant year, which was similar to the rate for placebo in Core (0.0065 per participant year; Table S3). Additional narrative detail of all deaths and a summary of SAEs occurring during Core + OLE can be found in Table S4 and Table S5, respectively. The most common SAEs in the Core + OLE were infusion-related reactions (1.2%) and ARIA-E (1.1%).

**Table 2:** Adverse events and ARIA in Clarity Core and Core + OLE

	Core		Core + OLE
	Placebo (*N* = 897) n/N (%)	Lecanemab (*N* = 898) n/N (%)	Lecanemab (*N* = 1612) n/N (%)
Any adverse event	735 (81.9)	798 (88.9)	1389 (86.2)
Deaths	7 (0.8)	6 (0.7)	16 (1.0)*
Serious adverse event (SAE)	101 (11.3)	126 (14.0)	241 (15.0)
SAE with ARIA-E	0	7 (0.8)	18 (1.1)
SAE with ARIA-H	0	2 (0.2)	10 (0.6)
SAE with infusion-related reactions	0	11 (1.2)	20 (1.2)
Treatment-related adverse event	197 (22.0)	401 (44.7)	721 (44.7)
Adverse event leading to drug withdrawal	26 (2.9)	62 (6.9)	124 (7.7)
ARIA-E	15/897 (1.7)	113/898 (12.6)	219/1612 (13.6)
ARIA-E by ApoE4 genotype			
ApoE4 noncarrier	1/286 (0.3)	15/278 (5.4)	32/496 (6.5)
ApoE4 carrier	14/611 (2.3)	98/620 (15.8)	187/1116 (16.8)
ApoE4 heterozygote	9/478 (1.9)	52/479 (10.9)	101/867 (11.6)
ApoE4 homozygote	5/133 (3.8)	46/141 (32.6)	86/249 (34.5)
Symptomatic ARIA-E	0	25/898 (2.8)	54/1612 (3.3)
ApoE4 noncarrier	0	4/278 (1.4)	8/496 (1.6)
ApoE4 carrier	0	21/620 (3.4)	46/1116 (4.1)
ApoE4 heterozygote	0	8/479 (1.7)	18/867 (2.1)
ApoE4 homozygote	0	13/141 (9.2)	28/249 (11.2)
Recurrent ARIA-E	1 (0.1)	28 (3.1)	46/1612 (2.9)
ApoE4 noncarrier	0/286 (0)	1/278 (0.4)	4/496 (0.8)
ApoE4 carrier	1/611 (0.2)	27/620 (4.4)	42/1116 (3.8)
ApoE4 heterozygote	0/478 (0)	7/479 (1.5)	18/867 (2.1)
ApoE4 homozygote	1/133 (0.8)	20/141 (14.2)	24/249 (9.6)
ARIA-H	80 (8.9)	152 (16.9)	298/1612 (18.5)
Microhemorrhage	68 (7.6)	126 (14.0)	258/1612 (16.0)
Superficial siderosis	21 (2.3)	50 (5.6)	96/1612 (6.0)
Intracerebral hemorrhage	1 (0.1)	5 (0.6)	8/1612 (0.5)
Symptomatic ARIA-H	2 (0.2)	11 (1.2)	27/1612 (1.7)
ARIA-H by ApoE4 genotype			
ApoE4 noncarrier, n/N (%)	11/286 (3.8)	32/278 (11.5)	59/496 (11.9)
ApoE4 carrier, n/N (%)	69/611 (11.3)	120/620 (19.4)	239/1116 (21.4)
ApoE4 heterozygote, n/N (%)	41/478 (8.6)	66/479 (13.8)	140/867 (16.1)
ApoE4 homozygote, n/N (%)	28/133 (21.1)	54/141 (38.3)	99/249 (39.8)
Isolated ARIA-H	69 (7.7)	78 (8.7)	146 (9.1)
Microhemorrhage	63 (7.0)	60 (6.7)	119 (7.4)
Superficial siderosis	13 (1.4)	23 (2.6)	39 (2.4)
Isolated intracerebral hemorrhage	1 (0.1)	4 (0.4)	5 (0.3)
Symptomatic isolated ARIA-H	2 (0.2)	4 (0.4)	6 (0.4)
Isolated ARIA-H by ApoE4 genotype			
ApoE4 noncarrier, n/N (%)	10/286 (3.5)	22/278 (7.9)	38/496 (7.7)
ApoE4 carrier, n/N (%)	59/611 (9.7)	56/620 (9.0)	108/1116 (9.7)
ApoE4 heterozygote, n/N (%)	35/478 (7.3)	39/479 (8.1)	76/867 (8.8)
ApoE4 homozygote, n/N (%)	24/133 (18.0)	17/141 (12.1)	32/249 (12.9)

*The 16 deaths included 6 from Core, 9 from OLE, and one death that occurred > 30 days after last dose

In Core + OLE, 86.2% of individuals in Core + OLE had at least one adverse event. (Table 2). Treatment-related adverse events and adverse events leading to drug discontinuation occurred in 44.7% and 7.7% of participants, respectively. The most common adverse events (> 10%) were infusion-related reactions (24.5%), ARIA-H (18.5% [microhemorrhages and superficial siderosis]), COVID-19 (14.7%), ARIA-E (13.6%), and headache (10.3%).

### ARIA-E and ARIA-H in Clarity AD

ARIA-E and ARIA-H were protocol-specified as adverse events of special interest in the lecanemab Clarity AD study and the top-level ARIA-E data for the Core study have been summarized previously [7]. ARIA-E and ARIA-H data for the Core and Core + OLE can be found in Table 2 and representative examples of imaging are shown in Figure S1. In Core + OLE, ARIA-E occurred in 13.6% of participants, with 3.3% symptomatic ARIA-E. When present, reported symptoms for participants with ARIA-E included headache, confusion, dizziness, vision changes, nausea, aphasia, weakness, or seizure. Focal neurologic deficits may also occur. Symptoms associated with ARIA-E usually resolve over time.

In Clarity AD, seizures were an infrequent symptom of ARIA-E or ARIA-H. In the Core + OLE, there were a total of 10 participants (0.6%) with seizures occurring concurrently with ARIA-E or ARIA-H (including ICH). Incidence of seizures unassociated with ARIA-E or ARIA-H events was similar (9/1612 [0.6%]); the incidence of unassociated seizures was the same between treatment groups: placebo 3/897 (0.3%) and lecanemab 3/898 (0.3%) in the Core study. Exposure-adjusted rates of death with concurrent ARIA (ARIA-E or ARIA-H; irrespective of whether ARIA-E or ARIA-H was the cause of death) were also similar between lecanemab in the Core + OLE and placebo in the Core (0.0013 [3 cases] and 0.0008 [1 case], respectively).

**Fig. 1:**
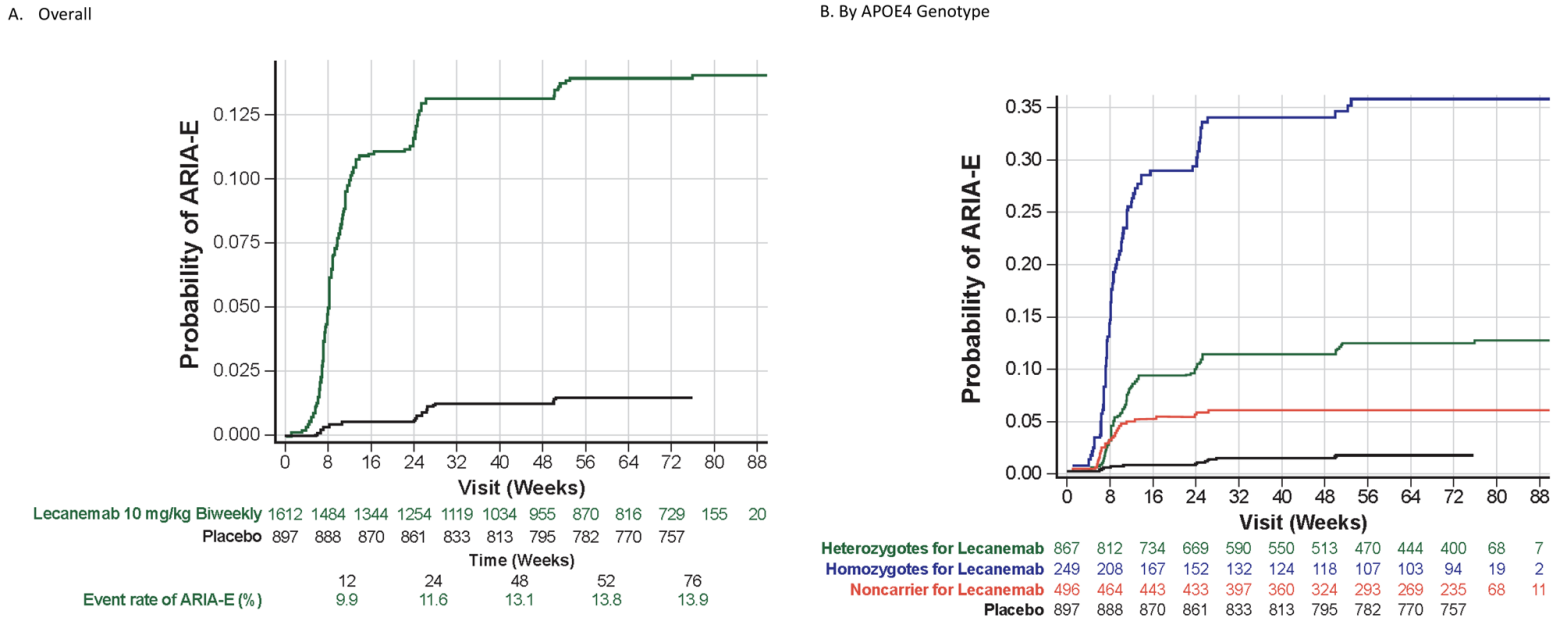
Timing of ARIA-E events (**A**) overall and (**B**) by APOE4 genotype for lecanemab in Core + OLE and placebo in Core

ARIA-E was more common in ApoE ε4 carriers, with highest frequency in homozygotes (noncarriers: 6.5%; heterozygotes: 11.6%; homozygotes: 34.5%). ARIA-E with lecanemab in Core + OLE generally occurred within the first 3 months (71%) or 6 months (92%; Fig. 1), generally resolving within 4 months of detection (81%), regardless of ApoE ε4 carrier status. Specifically, 60 of 111 participants (54%) were resolved by MRI at 90 days and 90 of 111 participants (81%) resolved by 120 days. Newly treated Core placebo participants had similar ARIA-E rates in the OLE as those for Core lecanemab participants.

A summary of radiographic and clinical severity of ARIA-E overall and by ApoE ε4 genotype can be found in Table 3. ARIA-E events were primarily mild-to-moderate radiographically (93/218; 88.5%) and asymptomatic (96.7%), regardless of ApoE ε4 genotype. Overall, 51% (49/96) participants with mild radiographic ARIA-E at onset did not worsen and continued dosing without drug interruption. Participants with mild radiographic ARIA-E who continued dosing resolved (3 months) in a similar time frame to those who discontinued dosing (4–5 months). Serious symptoms associated with ARIA were reported in 0.7% (6/898) of participants treated with lecanemab. Clinical symptoms associated with ARIA resolved in 79% (23/29) of participants during the period of observation.

**Table 3:** ARIA-E events: radiographic and clinical severity overall and by APOE4 genotype

			Radiographic severity (mild/moderate/severe)		Symptomatic ARIA-E	Symptomatic - Clinical Severity (mild/moderate/ severe)
**Core**	**Placebo** **(** ***N*** **= 897)**	**Lecanemab** **(** ***N*** **= 898)**	**Placebo** **(** ***N*** **= 897)**	**Lecanemab** **(** ***N*** **= 898)**	**Lecanemab** **(** ***N*** **= 898)**	**Lecanemab** **(** ***N*** **= 898)**
**ARIA-E**	15/897 (1.7%)	113/898 (12.6%)	9 / 6 / 0	37 / 66 / 9*	25/898 (2.8%)	10/12/3
**ApoE4+**	14/611 (2.3%)	98/620 (15.8%)	9 / 5 / 0	31 / 57 / 9*	21/620 (3.4%)	9/9/3
**Homozygote**	5/133 (3.8%)	46/141 (32.6%)	2 / 3 / 0	6 / 33 / 7	13/141 (9.2%)	5/7/1
**Heterozygote**	9/478 (1.9%)	52/479 (10.9%)	7 / 2 / 0	25 / 24 / 2*	8/479 (1.7%)	4/2/2
**ApoE4-**	1/286 (0.3%)	15/278 (5.4%)	0 / 1 / 0	6 / 9 / 0	4/278 (1.4%)	1/3/0
**Core + OLE**		**Lecanemab** **(** ***N*** **= 1612)**		**Lecanemab** **(** ***N*** **= 1612)**	**Lecanemab** **(** ***N*** **= 1612)**	**Lecanemab** **(** ***N*** **= 1612)**
**ARIA-E**		219/1612 (13.6%)		63 / 130 /25*	54/1612 (3.3%)	20/21/13
**ApoE4+**		187/1116 (16.8%)		50 / 111 /25*	46/1116 (4.1%)	17/17/12
**Homozygote**		86/249 (34.5%)		15 / 58 / 13	28/249 (11.2%)	10/13/5
**Heterozygote**		101/867 (11.6%)		35 / 53 / 12*	18/867 (2.1%)	7/4/7
**ApoE4-**		32/496 (6.5%)		13 / 19 / 0	8/496 (1.6%)	3/4/1

*One ARIA-E case has radiographic severity missing

The majority (> 95%) of participants had at least one post-ARIA cognitive assessment which was included in the primary analysis. Multiple analyses which included incorporating data after ARIA events (primary mixed model for repeated measures [MMRM]), censoring data after ARIA events, and imputing the data after ARIA events with placebo mean change show that ARIA did not adversely impact cognition or function. Results were highly statistically significant across all these analyses demonstrating that occurrence of ARIA events did not impact the efficacy of lecanemab. The impact of ARIA or ARIA-E on clinical efficacy was further evaluated by including ARIA or ARIA-E, as a [participant level and also as time-varying], covariate in the MMRM model for CDR-SB, Alzheimer’s disease Assessment Scale-Cognitive Subscale (ADAS-Cog14), and Alzheimer’s Disease Cooperative Study–Activities of Daily Living Scale for Mild Cognitive Impairment (ADCS MCI-ADL). ARIA or ARIA-E was not a significant covariate, and the estimated progression in those with or without ARIA or ARIA-E were similar.

**Fig. 2:**
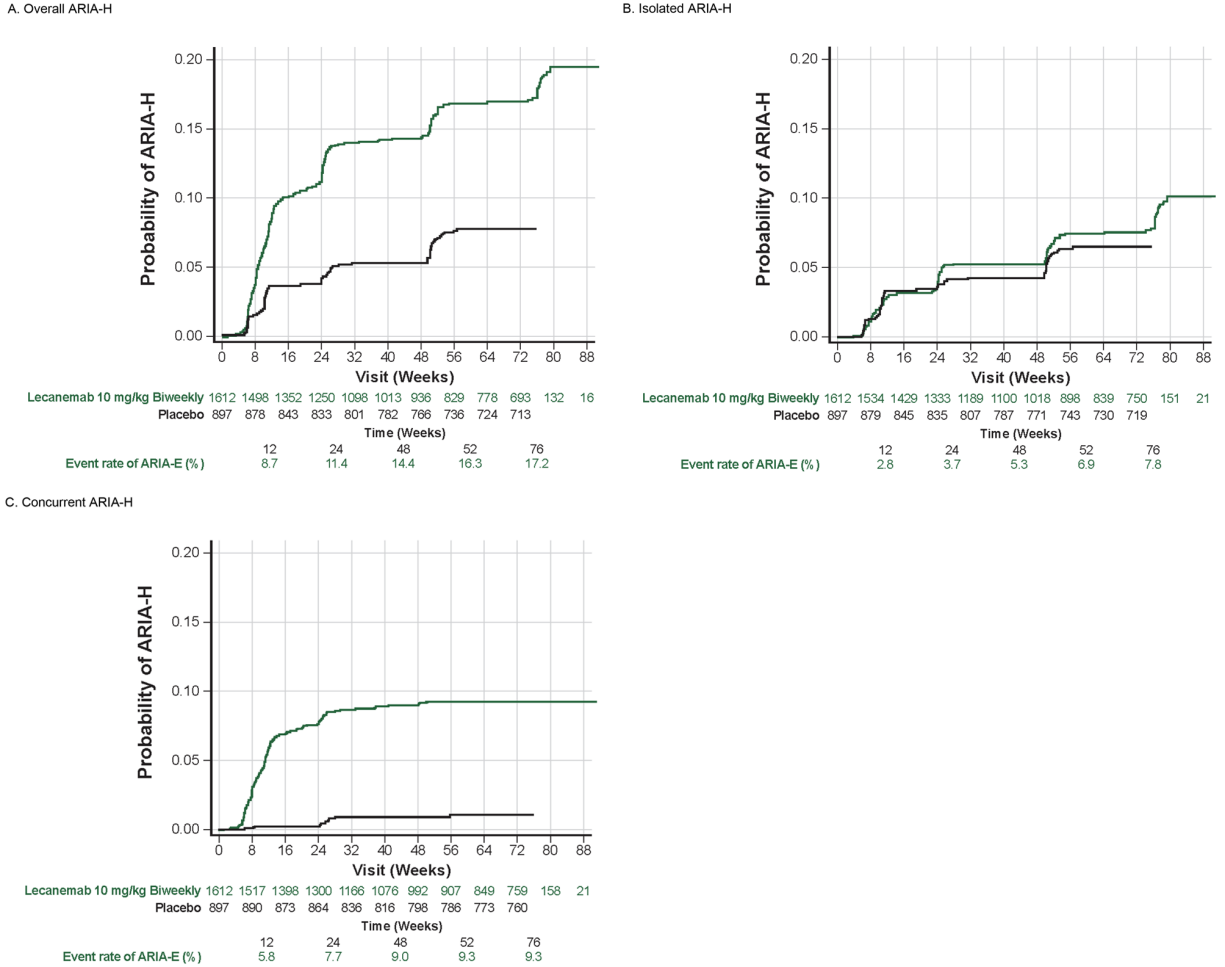
Timing of ARIA-H events (**A**) overall ARIA-H, (**B**) isolated ARIA-H, and (**C**) concurrent ARIA-H for lecanemab in Core + OLE and placebo in Core

The frequency of ARIA-H in Core + OLE was 18.5%, with symptomatic ARIA-H occurring in 1.7% of participants treated with lecanemab (Table 2). ARIA-H that did not occur together with ARIA-E (i.e., isolated ARIA-H) was 9.1% overall and 0.4% symptomatic. In the Core, isolated ARIA-H occurred at similar rates in the lecanemab and placebo groups [7] and results for Core + OLE were consistent. Timing of isolated ARIA-H events was at a steady rate across the treatment course at the same rate as placebo, while ARIA-H concurrent with ARIA-E, termed concurrent ARIA-H, occurred early in treatment. (Fig. 2). For ICH, there was likewise no clear relationship with timing of treatment initiation (Table 4). ApoE ε4 carrier status contributed to the incidence but not the timing of ARIA. Newly treated Core placebo participants had similar ARIA-H rates in the OLE as those for Core lecanemab participants.

**Table 4:** Intracerebral hemorrhage in lecanemab studies

Study	Treatment Group	Treatment Emergent	Anticoagulant	Antiplatelet	ASA	Isolated ICH or Concurrent with ARIA-E	APOE4 Genotype Status	Onset Day	Outcome	Symptomatic (Y/N)
301 Core	LEC-10BW	Y	N	Y	N	Concurrent	+/+	48	Not recovered/not resolved	N
301 Core	Placebo	N (stopped for ARIA 61 days before)	N	N	N	Concurrent	+/-	300	Recovering/Resolving	Y
301 Core	LEC-10BW	Y	N	N	N	Isolated	+/-	441	Not resolved	Y
301 Core	Placebo	Y	N	N	Y	Isolated	-/-	Unknown	Fatal	N
301 Core	LEC-10BW	N (stopped for ARIA 39 days before)	Y	N	N	Concurrent	+/-	85	Recovering/Resolving	Y
301 Core	LEC-10BW	Y	N	N	N	Isolated	-/-	439	Not resolved	Y
301 Core	LEC-10BW	Y	Y	N	Y	Isolated	+/+	175	Recovering/Resolving	N
301 Core	LEC-10BW	Y	N	N	N	Isolated	+/-	173	Recovering/Resolving	N
301 OLE	Core: PL OLE: LEC-10BW	Y	N	N	N	Isolated	+/+	OLE Day 276	Recovering/Resolving	Y
301 OLE	Core: PL OLE: LEC-10BW	Y	Y	N	N	Concurrent	+/-	OLE Day 58	Recovered/Resolved (Asymptomatic)	N
301 OLE	Core: PL OLE: LEC-10BW	Y	Y	N	N	Isolated	+/+	OLE Day ~ 30	Fatal	Y
301 OLE	Core: PL OLE: LEC-10BW	Y	Y	N	Y	Isolated	-/-	OLE Day 117	Fatal	Y

For severity, ARIA-H events were largely mild-to-moderate radiographically (240/298; 80.5%) and asymptomatic (271/298; 90.9%), with results consistent across ApoE ε4 genotypes. Most participants with mild radiographic ARIA-H did not worsen and could continue dosing without drug interruption (156/188 [83%]). Most cases of first mild radiographic ARIA-H (125/156, 80%) were stable at the next MRI. Severe ARIA-H in the Core + OLE was reported in 57 (3.5%) participants, mostly driven by any microhemorrhage event that resulted in a cumulative number greater than 10 microhemorrhages (47/1612 [2.9%]).

In Core + OLE, there was a low rate of ICH with lecanemab therapy (8/1612; 0.5%), which was higher than the rate observed in the Core placebo group (1/897; 0.1%). The rate of ICH for lecanemab-treated participants on anticoagulants was 2.7% (4/147) (Table 2). The background rate of ICH in AD participants on anticoagulation is not known but might be expected to be higher than in non-AD participants due to CAA; therefore, comparative risk is difficult to assess. There was no clear relationship of ICH to ApoE e4 status, baseline MRI, or timing of treatment.

Antiplatelet and anticoagulant utilization during Clarity AD is summarized in Table 5. In the Core, ARIA rates were slightly higher in the placebo group with anticoagulants, whereas ARIA rates were generally lower in participants who were on antiplatelet agents as well as those who were on anticoagulants. ARIA appeared less frequently in lecanemab-treated participants who were on antithrombotic medications, both antiplatelets and anticoagulants (participants who were on antithrombotic medications received similar overall lecanemab dose as those that were not). Results were generally consistent across ApoE ε4 genotypes and results in Core + OLE population were similar to Core results for lecanemab-treated participants. Logistic regression analyses were performed to evaluate the impact of various risk factors on ARIA-E, including baseline co-morbidities like hypertension, baseline amyloid status, APOE4 genotype, microhemorrhage at baseline, baseline disease stage etc. Based on these analyses, the only baseline risk factors identified for ARIA-E were ApoE ε4 genotype, microhemorrhage at baseline, and white matter abnormalities.

**Table 5:** Antiplatelet and anticoagulant use in Clarity AD in Core and Core + OLE

	ARIA-E			ARIA-H (microhemorrhage or superficial siderosis)			Intracerebral Hemorrhage		
	Core Placebo *N* = 897	Core Lecanemab *N* = 898	Core + OLE Lecanemab *N* = 1612	Core Placebo *N* = 897	Core Lecanemab *N* = 898	Core + OLE Lecanemab *N* = 1612	Core Placebo *N* = 897	Core Lecanemab *N* = 898	Core + OLE Lecanemab *N* = 1612
**No antiplatelet or anticoagulation at any time**	9/584 (1.5%)	74/545 (13.6%)	135/991 (13.6%)	49/584 (8.4%)	93/545 (17.1%)	183/991 (18.5%)	1/584 (0.2%)^1^	3/545 (0.6%)	4/991 (0.4%)
**Event post any antiplatelet (aspirin or non-aspirin)**	2/243 (0.8%)	30/271 (11.1%)	61/462 (13.2%)	22/243 (9.1%)	44/271 (16.2%)	85/462 (18.4%)	1/243 (0.4%)	1/271 (0.4%)	1/462 (0.2%)
**Event post any anticoagulation** **(alone or with antiplatelet)**	2/72 (2.8%)	4/79 (5.1%)	13/147 (8.8%)	7/72 (9.7%)	11/79 (13.9%)	21/147 (14.3%)	0/72 (0%)	2/79 (2.5%)^1^	4/147 (2.7%)^1^

^1^Includes one non-treatment emergent case on no antithrombotic and one on anticoagulation (event > 30 days after discontinuing study medication)

### Infusion reactions

Infusion-related reactions were limited in impact and most often occurred only at one infusion, without recurrence, regardless of whether prophylactic medications were administered. In the Core + OLE, infusion-related reactions occurred in 398/1612 (24.7%) of participants and in 142/714 [19.9%] of newly treated lecanemab participants in OLE. Infusion-related reactions were largely mild-to-moderate (96.5%) and largely occurred on the first dose (73%). Most participants (65.1%) with such reactions only had 1 infusion-related reaction, with common symptoms of infusion-related reactions including fever and flu-like symptoms (chills, generalized aches, feeling shaky, and joint pain), nausea, vomiting, hypotension, hypertension, and oxygen desaturation. Overall, 46.9% of participants received preventative (“prophylactic”) medications (e.g., acetaminophen, antihistamine, hydrocortisone) for an infusion after experiencing the first infusion-related reaction. Of the 173 participants who received at least 1 preventative medication, 68 (39.3%) had subsequent infusion reactions; 60.7% did not have subsequent infusion reactions. Of the 196 participants who did not receive a preventative medication, 64 (32.7%) had subsequent infusion reactions and 67.3% did not have subsequent infusion reactions. Severe infusion-related reactions (grade 3–4) occurred in 10/1612 (0.6%) participants, all of whom experienced the reaction with the first dose.

## Discussion

In this report, we have presented detailed results on the safety from the phase 3 Clarity AD Core + OLE, with a focus on ARIA. Overall, 1612 participants were included in Core + OLE dataset, which included 90% of individuals in the Core placebo group who enrolled into the Clarity AD OLE. In the Core + OLE, infusion reactions, ARIA-E, and ARIA-H were the most common adverse events of interest in the lecanemab group. ARIA-E and ARIA-H occur in the natural history of AD, as demonstrated by non-zero rates in the placebo group and are associated with the presence of CAA and ApoE e4 genotype status, with risks increased by anti-amyloid therapies [18]. The increased risk associated with the ApoE ε4 allele likely relates to the increased CAA known to occur with increased ApoE ε4 allele dosage. Microhemorrhages occur frequently in the natural history of AD, with rates as high as 21% over 18 months in ApoE ε4 homozygotes in the Core placebo group. The increased occurrence of ARIA-E seen with lecanemab treatment has been hypothesized to relate to the removal and disruption of amyloid in blood vessel walls and vascular remodeling [18], perivascular inflammation related to amyloid clearance, or possibly transiently increased CAA during clearance [21]. 

The safety of lecanemab in the Clarity AD Core + OLE is consistent with that in the Clarity AD Core and phase 2 Study, and rates of ARIA and symptomatic ARIA were low in comparison to those published for other anti-amyloid therapies (Table S6). To date, the post-marketing safety reports in the United States are also consistent with the safety profile observed with Clarity AD (Eisai Inc., Data on File). In the Core + OLE, ARIA-E occurs early in treatment (within first 3–6 months of treatment), is mostly asymptomatic (3.3% symptomatic ARIA-E) and resolves spontaneously regardless of radiographic severity (within 4 months of detection). Moreover, ARIA-E that is asymptomatic and radiographically mild can be dosed through without interruption. Increased number of ε4 alleles is a risk factor for ARIA-E; however, the clinical course of ARIA-E does not differ with number of ε4 alleles.

In the Core + OLE, the rate of ARIA-H (cerebral microhemorrhages and superficial siderosis) was 18.5% and of symptomatic ARIA-H was 1.7%. ARIA-H with lecanemab occurred concurrently with ARIA-E and in those cases, with the similar timing to the ARIA-E. The rate of isolated ARIA-H, occurring in the absence of ARIA-E, was similar in the placebo group in the Core and the lecanemab group in the Core + OLE. Isolated ARIA-H consisting of microhemorrhages and superficial siderosis, excluding ICH, occurred evenly throughout the treatment period, was almost always asymptomatic, and did not require alterations in dosing. Increased number of ε4 alleles is a risk factor for ARIA-H, but not for its clinical course.

Uncommonly, ARIA can be serious and life-threatening (SAE rate due to ARIA-E 1.1%, ARIA-H 0.6%). In the Core phase, there was one death due to intracerebral hemorrhage in a placebo participant. In lecanemab-treated participants in the Core + OLE phase, there were two deaths with concurrent intracerebral hemorrhage, and one with ARIA-E (Table S4). Exposure-adjusted death rates with or without concurrent ARIA or ICH overall were similar between lecanemab and placebo.

Lobar intracerebral hemorrhage can occur spontaneously in AD, typically attributed to CAA. Risk factors for ICH include ApoE ε4 genotype status, presence of microhemorrhages (an indicator of CAA), and anticoagulant medications. The background rate of cerebral macrohemorrhage in the placebo group of other AD clinical trials over 18 months was 0.4%, and in observational studies the annual rate of hemorrhagic stroke ranged from 0.0027 to 0.0052 person-years [22]. Across the Core + OLE, the rate of ICH with lecanemab therapy was 0.5% (0.00343 person-years). There was no clear relationship of ICH to ApoE ε4 genotype status, baseline MRI, or timing of treatment observed in our data. Antiplatelet and anticoagulant medications did not increase the risk of ARIA-E or ARIA-H in lecanemab treated participants. The number of intracerebral hemorrhage cases was small, limiting risk assessment of concomitant use of anticoagulants.

Enhanced clinical vigilance for ARIA is recommended during the first 14 weeks when treating with an anti-amyloid therapy; specific monitoring guidance is included in the approved lecanemab prescribing information [23]. As noted in the lecanemab label, if a participant experiences symptoms suggestive of ARIA, clinical evaluation should be performed, including MRI scanning if indicated. Although ARIA-E is most likely to occur early during therapy, late events can occur, so vigilance for symptoms is always recommended during treatment. Since intracerebral hemorrhage has been observed in participants taking lecanemab, additional caution should be exercised when considering the use of lecanemab with anticoagulants or a thrombolytic agent.

For lecanemab, the occurrence of ARIA-E is dose-dependent and increased incidence is associated with the ε4 allele of ApoE gene, which is a similar profile to that observed for bapineuzumab, donanemab, gantenerumab, and aducanumab. Indirect comparison between trials, would suggest that lecanemab, even though there is no titration, has lower ARIA-E than some of the other published Aβ immunotherapies, including those of aducanumab and donanemab trials (Table S6) [7,18,22,24–27]. The variance in incidence rates and timing of ARIA across these treatments may be related to differences in antibody binding profiles to soluble aggregated amyloid species, amyloid plaques, and vascular amyloid.

Future research on lecanemab will include longer-term data, including updated data from ongoing OLE for the lecanemab phase 2 and Clarity AD. While ARIA and infusion reactions tend to occur early in treatment, these data will give additional insight into ICH risk and risks with longer term exposure. In addition, research on alternate lecanemab formulations will explore whether subcutaneous administration can enhance the lecanemab safety profile (e.g., ARIA rates, infusion reactions, etc.).

In summary, lecanemab demonstrated reduction in brain amyloid accompanied by a consistent reduction of clinical decline across several clinical endpoints in participants with early AD. Lecanemab was generally well-tolerated, with the most common adverse events being infusion-related reactions, ARIA-H, ARIA-E, and headache. In the Core study, ARIA-E occurred more frequently in participants treated with lecanemab than placebo but was largely radiographically mild-to-moderate, and generally occurred within the first 3–6 months of treatment. ARIA was more common in ApoE ε4 carriers and most common in ApoE ε4 homozygous participants. Infusion-related reaction, ARIA-E, and rare ICH are important adverse events that can be seen with lecanemab treatment. Clinicians, participants, and caregivers should understand the incidence, monitoring, and management of these events for optimal patient care. [27,28]

### Electronic supplementary material

Below is the link to the electronic supplementary material.

Supplementary Material 1

## Data Availability

Not available.
