# Correction: Updated safety results from phase 3 lecanemab study in early Alzheimer’s disease

**DOI:** 10.1186/s13195-024-01507-7

**Published:** 2024-07-10

**Authors:** Lawrence S. Honig, Marwan N. Sabbagh, Christopher H. van Dyck, Reisa A. Sperling, Steven Hersch, Andre Matta, Luigi Giorgi, Michelle Gee, Michio Kanekiyo, David Li, Derk Purcell, Shobha Dhadda, Michael Irizarry, Lynn Kramer

**Affiliations:** 1 Columbia University Irving Medical Center, NYS Center of Excellence for Alzheimer’s Disease, Taub Institute for Research on Alzheimer’s Disease and the Aging Brain, Gertrude H. Sergievsky Center (PH19), & Department of Neurology, Columbia University Vagelos College of Physicians & Surgeons, 630 West 168th Street (P&S UNIT 16), New York, NY 10032-3795 USA; 2 Barrow Neurological Institute, Phoenix, AZ 85013 USA; 3 Yale School of Medicine, New Haven, CT USA; 4 Brigham and Women’s Hospital, Massachusetts General Hospital, Harvard Medical School, Boston, MA USA; 5 Eisai Inc., Nutley, NJ USA; 6 Eisai Co., Ltd, Hatfield, UK; 7 Clario, Philadelphia, PA USA

**Correction:** ***Alz Res Therapy*** **16, 105 (2024)**

10.1186/s13195-024-01441-8

Following publication of the original article [1], several typographical errors were observed:

- In Table 2, recurrent ARIA-E for ApoE carriers should be 3.8% (not 11.7%).
- In Table 2, there were several incorrect indentations for several rows (old and updated versions of Table 2 are presented below).
- There are two places on page 6 where ‘ARIA-E’ should be ‘ARIA’.
- In Table 5, the asterisk in right column should be a superscript ‘1’. The footnote should have an end parenthesis at end of the footnote sentence below the table.

Moreover, Supplementary material 1 (.docx) has been replaced with its pdf version with modification in Table S4 - ID21 (the participant should be “male” instead of “female”).

The old version of Table 2:

**Table 2:** Adverse events and ARIA in Clarity Core and Core + OLE

	Core		Core + OLE
	**Placebo (N = 897) n/N (%)**	**Lecanemab (N = 898) n/N (%)**	Lecanemab (N = 1612) n/N (%)
Any adverse event	735 (81.9)	798 (88.9)	1389 (86.2)
Deaths	7 (0.8)	6 (0.7)	16 (1.0)*
Serious adverse event (SAE)	101 (11.3)	126 (14.0)	241 (15.0)
SAE with ARIA-E	0	7 (0.8)	18 (1.1)
SAE with ARIA-H	0	2 (0.2)	10 (0.6)
SAE with infusion-related reactions	0	11 (1.2)	20 (1.2)
Treatment-related adverse event	197 (22.0)	401 (44.7)	721 (44.7)
Adverse event leading to drug withdrawal	26 (2.9)	62 (6.9)	124 (7.7)
ARIA-E	15/897 (1.7)	113/898 (12.6)	219/1612 (13.6)
ARIA-E by ApoE4 genotype			
ApoE4 noncarrier	1/286 (0.3)	15/278 (5.4)	32/496 (6.5)
ApoE4 carrier	14/611 (2.3)	98/620 (15.8)	187/1116 (16.8)
ApoE4 heterozygote	9/478 (1.9)	52/479 (10.9)	101/867 (11.6)
ApoE4 homozygote	5/133 (3.8)	46/141 (32.6)	86/249 (34.5)
Symptomatic ARIA-E	0	25/898 (2.8)	54/1612 (3.3)
ApoE4 noncarrier	0	4/278 (1.4)	8/496 (1.6)
ApoE4 carrier	0	21/620 (3.4)	46/1116 (4.1)
ApoE4 heterozygote	0	8/479 (1.7)	18/867 (2.1)
ApoE4 homozygote	0	13/141 (9.2)	28/249 (11.2)
Recurrent ARIA-E	1 (0.1)	28 (3.1)	46/1612 (2.9)
ApoE4 noncarrier	0/286 (0)	1/278 (0.4)	4/496 (0.8)
ApoE4 carrier	1/611 (0.2)	27/620 (4.4)	42/1116 (11.7)
ApoE4 heterozygote	0/478 (0)	7/479 (1.5)	18/867 (2.1)
ApoE4 homozygote	1/133 (0.8)	20/141 (14.2)	24/249 (9.6)
ARIA-H	80 (8.9)	152 (16.9)	298/1612 (18.5)
Microhemorrhage	68 (7.6)	126 (14.0)	258/1612 (16.0)
Superficial siderosis	21 (2.3)	50 (5.6)	96/1612 (6.0)
Intracerebral hemorrhage	1 (0.1)	5 (0.6)	8/1612 (0.5)
Symptomatic ARIA-H	2 (0.2)	11 (1.2)	27/1612 (1.7)
ARIA-H by ApoE4 genotype			
ApoE4 noncarrier, n/N (%)	11/286 (3.8)	32/278 (11.5)	59/496 (11.9)
ApoE4 carrier, n/N (%)	69/611 (11.3)	120/620 (19.4)	239/1116 (21.4)
ApoE4 heterozygote, n/N (%)	41/478 (8.6)	66/479 (13.8)	140/867 (16.1)
ApoE4 homozygote, n/N (%)	28/133 (21.1)	54/141 (38.3)	99/249 (39.8)
Isolated ARIA-H	69 (7.7)	78 (8.7)	146 (9.1)
Microhemorrhage	63 (7.0)	60 (6.7)	119 (7.4)
Superficial siderosis	13 (1.4)	23 (2.6)	39 (2.4)
Isolated intracerebral hemorrhage	1 (0.1)	4 (0.4)	5 (0.3)
Symptomatic isolated ARIA-H	2 (0.2)	4 (0.4)	6 (0.4)
Isolated ARIA-H by ApoE4 genotype			
ApoE4 noncarrier, n/N (%)	10/286 (3.5)	22/278 (7.9)	38/496 (7.7)
ApoE4 carrier, n/N (%)	59/611 (9.7)	56/620 (9.0)	108/1116 (9.7)
ApoE4 heterozygote, n/N (%)	35/478 (7.3)	39/479 (8.1)	76/867 (8.8)
ApoE4 homozygote, n/N (%)	24/133 (18.0)	17/141 (12.1)	32/249 (12.9)

*The 16 deaths included 6 from Core, 9 from OLE, and one death that occurred > 30 days after last dose

The updated version of Table 2:

**Table 2:** Adverse events and ARIA in Clarity Core and Core + OLE

	Core		Core + OLE
	**Placebo (N = 897) n/N (%)**	**Lecanemab (N = 898) n/N (%)**	Lecanemab (N = 1612) n/N (%)
Any adverse event	735 (81.9)	798 (88.9)	1389 (86.2)
Deaths	7 (0.8)	6 (0.7)	16 (1.0)*
Serious adverse event (SAE)	101 (11.3)	126 (14.0)	241 (15.0)
SAE with ARIA-E	0	7 (0.8)	18 (1.1)
SAE with ARIA-H	0	2 (0.2)	10 (0.6)
SAE with infusion-related reactions	0	11 (1.2)	20 (1.2)
Treatment-related adverse event	197 (22.0)	401 (44.7)	721 (44.7)
Adverse event leading to drug withdrawal	26 (2.9)	62 (6.9)	124 (7.7)
ARIA-E	15/897 (1.7)	113/898 (12.6)	219/1612 (13.6)
ARIA-E by ApoE4 genotype			
ApoE4 noncarrier	1/286 (0.3)	15/278 (5.4)	32/496 (6.5)
ApoE4 carrier	14/611 (2.3)	98/620 (15.8)	187/1116 (16.8)
ApoE4 heterozygote	9/478 (1.9)	52/479 (10.9)	101/867 (11.6)
ApoE4 homozygote	5/133 (3.8)	46/141 (32.6)	86/249 (34.5)
Symptomatic ARIA-E	0	25/898 (2.8)	54/1612 (3.3)
ApoE4 noncarrier	0	4/278 (1.4)	8/496 (1.6)
ApoE4 carrier	0	21/620 (3.4)	46/1116 (4.1)
ApoE4 heterozygote	0	8/479 (1.7)	18/867 (2.1)
ApoE4 homozygote	0	13/141 (9.2)	28/249 (11.2)
Recurrent ARIA-E	1 (0.1)	28 (3.1)	46/1612 (2.9)
ApoE4 noncarrier	0/286 (0)	1/278 (0.4)	4/496 (0.8)
ApoE4 carrier	1/611 (0.2)	27/620 (4.4)	42/1116 (3.8)
ApoE4 heterozygote	0/478 (0)	7/479 (1.5)	18/867 (2.1)
ApoE4 homozygote	1/133 (0.8)	20/141 (14.2)	24/249 (9.6)
ARIA-H	80 (8.9)	152 (16.9)	298/1612 (18.5)
Microhemorrhage	68 (7.6)	126 (14.0)	258/1612 (16.0)
Superficial siderosis	21 (2.3)	50 (5.6)	96/1612 (6.0)
Intracerebral hemorrhage	1 (0.1)	5 (0.6)	8/1612 (0.5)
Symptomatic ARIA-H	2 (0.2)	11 (1.2)	27/1612 (1.7)
ARIA-H by ApoE4 genotype			
ApoE4 noncarrier, n/N (%)	11/286 (3.8)	32/278 (11.5)	59/496 (11.9)
ApoE4 carrier, n/N (%)	69/611 (11.3)	120/620 (19.4)	239/1116 (21.4)
ApoE4 heterozygote, n/N (%)	41/478 (8.6)	66/479 (13.8)	140/867 (16.1)
ApoE4 homozygote, n/N (%)	28/133 (21.1)	54/141 (38.3)	99/249 (39.8)
Isolated ARIA-H	69 (7.7)	78 (8.7)	146 (9.1)
Microhemorrhage	63 (7.0)	60 (6.7)	119 (7.4)
Superficial siderosis	13 (1.4)	23 (2.6)	39 (2.4)
Isolated intracerebral hemorrhage	1 (0.1)	4 (0.4)	5 (0.3)
Symptomatic isolated ARIA-H	2 (0.2)	4 (0.4)	6 (0.4)
Isolated ARIA-H by ApoE4 genotype			
ApoE4 noncarrier, n/N (%)	10/286 (3.5)	22/278 (7.9)	38/496 (7.7)
ApoE4 carrier, n/N (%)	59/611 (9.7)	56/620 (9.0)	108/1116 (9.7)
ApoE4 heterozygote, n/N (%)	35/478 (7.3)	39/479 (8.1)	76/867 (8.8)
ApoE4 homozygote, n/N (%)	24/133 (18.0)	17/141 (12.1)	32/249 (12.9)

*The 16 deaths included 6 from Core, 9 from OLE, and one death that occurred > 30 days after last dose

The original article [1] has been updated.
