# Supplementary material for: Updated safety results from phase 3 lecanemab study in early Alzheimer’s disease
Source: Alzheimers Res Ther. 2024 May 10;16:105. doi: 10.1186/s13195-024-01441-8 (PMC11084061; doi:10.1186/s13195-024-01441-8)

Supplementary Appendix

Table S1. ARIA-E MRI Classification Criteria

Radiographic Severity		
**Mild**	**Moderate**	**Severe**
FLAIR hyperintensity confined to sulcus, and/or cortex/subcortex white matter in one location <5 cm	FLAIR hyperintensity 5-10 cm in single greatest dimensions, or more than 1 site of involvement each measuring <10 cm	FLAIR hyperintensity >10 cm with associated gyral swelling and sulcal effacement. One or more separate/independent sites of involvement may be noted

ARIA-E = amyloid-related imaging abnormality – edema/effusion; FLAIR = Fluid Attenuating Inversion Recovery; MRI = magnetic resonance imaging.

Table S2. Deaths and SAEs by APOE4 Genotype in Clarity AD Core and Core + OLE

CORE								
	Placebo (All) (n=897) n (%)	Lecanemab (All) (n=898) n (%)	Placebo Noncarriers (n=286) n (%)	Lecanemab Noncarriers (n=278) n (%)	Placebo Heterozygotes (n=478) n (%)	Lecanemab Heterozygotes (n=479) n (%)	Placebo Homozygotes (n=133) n (%)	Lecanemab Homozygotes (n=141) n (%)
**Deaths***	7 (0.8)	6 (0.7)	5 (1.7)	5 (1.8)	2 (0.4)	1 (0.2)	0	0
**Serious adverse event (SAE)**	101 (11.3)	126 (14.0)	34 (11.9)	41 (14.7)	52 (10.9)	71 (14.8)	15 (11.3)	14 (9.9)
**SAE with ARIA-E**	0	7 (0.8)	0	2 (0.7)	0	3 (0.6)	0	2 (1.4)
**SAE with ARIA-H**	0	2 (0.2)	0	1 (0.4)	0	0	0	1 (0.7)
**SAE with ICH**	1 (0.1)	3 (0.3)	1 (0.3)	1 (0.4)	0	1 (0.2)	0	1 (0.7)
**SAE with infusion-related reactions**	0	11 (1.2)	0	1 (0.4)	0	10 (2.1)	0	0
**SAE without ARIA, ICH or infusion-related reactions**	101 (11.3)	111 (12.4)	34 (11.9)	38 (13.7)	52 (10.9)	63 (13.2)	15 (11.3)	10 (7.1)
**CORE + OLE**								
		Lecanemab (All) (n=1612) n (%)		Lecanemab Noncarriers (n=496) n (%)		Lecanemab Heterozygotes (n=867) n (%)		Lecanemab Homozygotes (n=249) n (%)
**Deaths***		16 (1.0)**		9 (1.8)		5 (0.6)		2 (0.8)
**Serious adverse event (SAE)**		241 (15.0)		69 (13.9)		132 (15.2)		40 (16.1)
**SAE with ARIA-E**		18 (1.1)		4 (0.8)		6 (0.7)		8 (3.2)
**SAE with ARIA-H**		10 (0.6)		2 (0.4)		3 (0.3)		5 (2.0)
**SAE with ICH**		6 (0.4)		2 (0.4)		2 (0.2)		2 (0.8)
**SAE with infusion-related reactions**		20 (1.2)		5 (1.0)		14 (1.6)		1 (0.4)
**SAE without ARIA, ICH or infusion-related reactions**		205 (12.7)		61 (12.3)		115 (13.3)		29 (11.6)

ICH, intracerebral hemorrhage
*Cause of deaths in Core placebo group: myocardial infarction, COVID-19, COVID-19 pneumonia, left occipital intracerebral hemorrhage (ARIA-H) symptomatic, Possible Seizure and possible cerebrovascular accident (unknown), acute multifocal ich post tPA fatal car accident. Cause of death in Core lecanemab group: death, cerebrovascular accident, myocardial infarction, respiratory failure, Diabetic ketoacidosis, metastases to meninges, Cerebrovascular accident, COVID-19, Cardiac failure acute. **The 16 deaths included 6 from Core, 9 from OLE, and one death that occurred >30 days after last dose.

Table S3. Exposure-Adjusted Death Rates Overall and by ApoE4 Carrier Status.

	**Placebo (Double-Blind) N = 897**	**Lecanemab (Double-Blind) N = 898**	**Lecanemab (OLE) N = 1385**	**Lecanemab (Double-Blind + OLE) N = 1612**
**Overall**				
Number of deaths, n	8	7*	9	16**
Rate of death, per participant year	0.0065	0.0059	0.0078	0.0069
Number of death with concurrent ARIA or ICH irrespective of ARIA or ICH being cause of death, n	1	0	3	3
Rate of death with concurrent ARIA irrespective of ARIA being cause of death, per participant year	0.0008	0	0.0026	0.0013
**by APOE4 Genotype**				
Number of death<br>Noncarrier / heterozygote / homozygote, n	5 / 3 / 0	5 / 2 / 0	4 / 3 / 2	9 / 5 / 2
Rate of death<br>Noncarrier / heterozygote / homozygote, per participant year	0.0130 /0.0045/0	0.0139/0.0031/0	0.0101/0.0050/0.0129	0.0119 / 0.0040 / 0.0059
Number of death with concurrent ARIA irrespective of ARIA being cause of death<br>Noncarrier heterozygote / homozygote, n	1 / 0 / 0	0 / 0 / 0	1 / 0 / 2	1 / 0 / 2
Rate of death with concurrent ARIA irrespective of ARIA being cause of death<br>Noncarrier / heterozygote / homozygote, per participant year	0.0026 / 0 / 0	0 / 0 / 0	0.0025 / 0 / 0.0129	0.0013 / 0 / 0.0059

All deaths occurred during study, with data cut of 01 Dec 2022
OLE = Open-Label Extension. *includes one death that occurred >30 days after last dose. **The 16 deaths included 6 from Core, 9 from OLE, and one death that occurred >30 days after last dose.

Table S4. Additional Narrative Detail on Deaths from Clarity AD Core and OLE for Participants Receiving Lecanemab or Placebo.

ID	AE Start Study Day	Study Day of Death	Days Since Last Dose to Death	Fatal Event (Verbatim Term)	Other Relevant Details
**Double-blind Placebo**					
1	462	462	7	Unknown cause	A 90-year-old participant with sudden death of unknown cause. Long cardiac history with previous myocardial infarctions, bundle branch block, aortic stenosis, angina pectoris and multiple concomitant medications for cardiac history. Complaints prior to death of shortness of breath and not feeling well.
2	4	4	4	Acute respiratory failure	An 82-year-old participant, no other relevant details.
3	36	37	5	Heart attack	A 79-year-old participant, no other relevant details.
4	546	607	76	Bone metastases	A 75-year-old participant, no other relevant details.
5	Unknown	402	48	Intracerebral hemorrhage	This was a 72-year-old APOE4 noncarrier who received placebo with their last dose of study drug on Day 355. On Day 361 the participant presented with confusional state, was hospitalized, and then discharged on Day 362. The participant experienced an intracranial hemorrhage on an unknown date. On Day 402 the participant died due to intracranial hemorrhage.
6	404	416	24	COVID-19	An 86-year-old participant, no other relevant details.
7	71	319	249	Pancreatic cancer	An 85-year-old participant, no other relevant details.
8	301	301	49	Cardiopulmonary arrest[a]	A 79-year-old participant, no other relevant details.
**Double-blind Lecanemab**					
9	434	434	14	Unknown cause	An 85-year-old participant, sudden death in a setting of no known serious comorbidities.
10	263	282	46	Stroke, acute, symptomatic	A 79-year-old participant, no other relevant details.

ID	AE Start Study Day	Study Day of Death	Days Since Last Dose to Death	Fatal Event (Verbatim Term)	Other Relevant Details
11	230	230	11	Suspected myocardial infarction	A 70-year-old participant, significant medical history included diabetes, hypertension, coronary artery disease, prior myocardial infarction, and cardiac arrythmia and conduction defects. Experienced dyspnea, collapsed, and died suddenly.
12	351	405	76	Respiratory failure	An 88-year-old participant, significant medical history included coronary artery disease, diabetes, hyperlipidemia, hypertension, chronic obstructive pulmonary disease, cardiac dysrhythmia including atrioventricular block with left ventricular hypertrophy. Participant experienced new onset atrial fibrillation with rapid response and a cardiac arrest soon afterwards during an AVM ablation procedure. Immediate sequelae included respiratory failure, intubation, iatrogenic pneumonia, and pulmonary embolism and edema. Participant received a tracheostomy and was transferred to an acute long-term care hospital where she expired.
13	534	563	45	Lymphomatous meningitis	A 77-year-old participant, no other relevant details.
14	375	402	23	Respiratory tract infection SARS-CoV19	A 76-year-old participant, no other relevant details.
15	526	526	36	DKA (Diabetic ketoacidosis)[b]	A 79-year-old participant, no other relevant details.
**Open-Label Extension[c]**					
16	11	11	11	Myocardial infarction	A 78-year-old participant, no other relevant details.
17	103	129	32	COVID-19 with pneumonia	A 68-year-old participant, no other relevant details.

ID	AE Start Study Day	Study Day of Death	Days Since Last Dose to Death	Fatal Event (Verbatim Term)	Other Relevant Details
18	515	519	15	COVID-19	An 85-year-old participant, no other relevant details.
19	188	188	16	Fatal car accident	A 64-year-old participant, no other relevant details.
20	777	777	20	Cardiac failure acute	An 81-year-old participant, no other relevant details.
21	116	143	46	Left occipital intracerebral hemorrhage (ARIA-H) symptomatic	This was an 85-year-old APOE4 noncarrier male who received placebo in the double-blind and then 3 doses of lecanemab in the OLE. The participant had falls, COVID-19, pneumonia, atrial fibrillation and had a 1.6 cm intracerebral hemorrhage while on apixaban. The apixaban was discontinued and the participant died after a myocardial infarction. Autopsy showed minimal-mild CAA, subacute ICH, subacute to chronic SAH with history of falls, and concluded a terminal cardiopulmonary event was the likely cause of death.
22	38	43	13	Possible Seizure (unknown) (suspected) and possible cerebrovascular accident (unknown) (suspected)	This was a 77-year-old APOE4 homozygous female who received placebo in the double-blind and then 3 doses of lecanemab in the OLE. The participant had acute hemiparesis, possible seizure, and hypoxia in a setting of new onset atrial fibrillation and subsequent aspiration pneumonia and death. MRI showed radiographically severe ARIA-E with 51 microhemorrhages, autopsy showed AD severe CAA vasculitic changes and death from arteriosclerotic heart disease and ARDS
23	33	37	9	Acute multifocal ICH (Intracerebral hemorrhage) POST TPA	This was a 63-year-old APOE4 homozygous carrier who received placebo in the double-blind and then 3 doses of lecanemab in the OLE. The participant had multiple intracerebral hemorrhages minutes after receiving TPA for left middle cerebral artery occlusion. The participant had severe cerebral amyloid angiopathy and histiocytic vasculitis by autopsy.

ID	AE Start Study Day	Study Day of Death	Days Since Last Dose to Death	Fatal Event (Verbatim Term)	Other Relevant Details
24	695	696	11	Symptomatic suspected cerebral vascular accident	A 72-year-old participant who received lecanemab in the double-blind Study and OLE. Participant suspected to have experienced symptomatic cerebral vascular accident which was classified as severe in severity and serious (fatal). Participant presented with mild dysarthria and 2-day history of diarrhea; was alert, conscious and cooperating. Physical exam revealed mild pain in epigastric region and left hypochondrium region and no facial or limbs weakness. Brain CT showed modest atrophic enlargement of supratentorial ventricular system and periventricular white matter hypodensity from chronic flow deficit. Participant later found nonresponsive to verbal stimuli and painful stimulus; treated with methylprednisolone, bicarbonate, dopamine and IV fluids; repeat CT showed no new ischemic lesions. Cause of death reported as cardiorespiratory arrest. Death considered possibly related to study drug by Investigator.

All deaths occurred during study, with data cut of 01 Dec 2022.
a: Event occurred 49 days since last dose, not included in treatment-emergent adverse event summaries. b: Event occurred 36 days since last dose, not included in treatment-emergent adverse event summaries. c: Study Day is based on first infusion date of lecanemab in double blind phase or OLE Phase.

Table S5. Serious Adverse Events from Clarity AD Core Phase and the Open-Label Extension

MedDRA Preferred Term	Clarity AD Trial		
	Core, Double-Blind Phase		Cumulative (Core + OLE)
	Placebo (N = 897) n(%)	Lecanemab (N = 898) n(%)	Lecanemab (N = 1612) n(%)
Participants with any serious adverse events	101 (11.3)	126 (14.0)	241 (15.0)
Infusion-related reaction	0	11 (1.2)	20 (1.2)
Amyloid related imaging abnormality-oedema/effusion	0	7 (0.8)	18 (1.1)
Atrial fibrillation	3 (0.3)	6 (0.7)	8 (0.5)
Syncope	1 (0.1)	6 (0.7)	8 (0.5)
Angina pectoris	0	6 (0.7)	8 (0.5)
Diverticulitis	1 (0.1)	4 (0.4)	4 (0.2)
Non-cardiac chest pain	0	4 (0.4)	5 (0.3)
Pneumonia	3 (0.3)	3 (0.3)	9 (0.6)
Subdural haematoma	3 (0.3)	3 (0.3)	5 (0.3)
Inguinal hernia	2 (0.2)	3 (0.3)	4 (0.2)
Hip fracture	2 (0.2)	3 (0.3)	3 (0.2)
Transient ischaemic attack	2 (0.2)	3 (0.3)	5 (0.3)
Fall	1 (0.1)	3 (0.3)	5 (0.3)
Cerebral haemorrhage	0	3 (0.3)	6 (0.4)
Acute respiratory failure	3 (0.3)	2 (0.2)	3 (0.2)
Osteoarthritis	3 (0.3)	2 (0.2)	4 (0.2)
Femoral neck fracture	1 (0.1)	2 (0.2)	3 (0.2)
Acute myocardial infarction	0	2 (0.2)	6 (0.4)
ARIA-H	0	2 (0.2)	9 (0.6)
COVID-19 pneumonia	0	2 (0.2)	4 (0.2)
Hyponatraemia	0	2 (0.2)	4 (0.2)
Ankle fracture	3 (0.3)	1 (0.1)	1 (0.1)
Prostate cancer	3 (0.3)	1 (0.1)	1 (0.1)
Pulmonary embolism	3 (0.3)	1 (0.1)	1 (0.1)
Urinary tract infection	1 (0.1)	1 (0.1)	7 (0.4)
Confusional state	3 (0.3)	0	0
Spinal compression fracture	3 (0.3)	0	0

ARIA-H, Amyloid related imaging abnormality-microhaemorrhages and haemosiderin deposits.
Note: This table includes an update of those who are in the ongoing Clarity AD open-label extension (OLE). Event terms in this table are per MedDRA ICH. The Cumulative Core + Open-Label Extension) column includes the cumulative serious adverse event data for the 898 participants who received lecanemab in the core in addition to all of their respective serious adverse events from the OLE as well as the 714 participants who were not on drug in the core and converted to lecanemab in the OLE (cutoff as of December 1, 2022).

Table S6. Cross-Study Comparison of ARIA in Various Phase 3 Studies of Approved or Active Development Anti-Amyloid Monoclonal Antibodies**

	**Aducanumab (ADU) Phase 3 Trial[1-3]**		**Lecanemab (LEC) Phase 3 Trial[4-5]**		**Donanemab (DON) Phase 3 Trial[6]**	
	**ADU****	Placebo	**LEC**	Placebo	**DON**	Placebo
**ARIA-E**						
Overall	**35.2%**	2.7%	**12.6%**	1.7%	**24.0%**	1.9%
Symptomatic	**9.1%**	0.3%	**2.8%**	0	**6.1%**	0.1%
ARIA-E by ApoE4 genotype						
ApoE4 noncarrier	**20.3%**	3.9%	**5.4%**	0.3%	**15.7%**	0.8%
ApoE4 heterozygote	**35.9%**	†	**10.9%**	1.9%	**22.8%**	1.9%
ApoE4 homozygote	**66.0%**	†	**32.6%**	3.8%	**40.6%**	3.4%
**ARIA-H**						
Overall (microhemorrhage and superficial siderosis)	**28.2%**	8.6%	**16.9%**	8.9%	**31.4%**	13.6%
Microhemorrhage	**19.1%**	6.6%	**14.0%**	7.6%	**26.8%**	12.5%
Superficial siderosis	**14.7%**	2.2%	**5.6%**	2.3%	**15.7%**	3.0%
Intracerebral hemorrhage	**0.3%**	0.4%	**0.6%**	0.1%	**0.4%**	0.2%
Isolated ARIA-H	**6.0%**	7.5%	**8.7%**	7.8%	**12.7%**	12.4%

*Data presented in this table are from 3 different randomized controlled phase 3 trials with different study populations and methodologies. This table is intended as a reference and not for direct cross-study comparisons.
**Numbers are based on ARIA in the 10 mg/kg (FDA licensed) dose group only.
† ARIA-H for ApoE4 carriers for the placebo group was 2.2%. The published reports do not break down by ApoE4 carrier subgroups.

1. Budd Haeberlein S, Aisen PS, Barkhof F, et al. Two Randomized Phase 3 Studies of Aducanumab in Early Alzheimer's Disease. J Prev Alzheimers Dis. 2022;9:197-210.
2. Salloway S, Chalkias S, Barkhof F, et al. Amyloid-Related Imaging Abnormalities in 2 Phase 3 Studies Evaluating Aducanumab in Patients With Early Alzheimer Disease. JAMA Neurol. 2022 Jan 1;79(1):13-21.
3. Aducanumab-avwa prescribing information. Available at: https://www.accessdata.fda.gov/drugsatfda_docs/label/2021/761178s000lbl.pdf Accessed July 23, 2023.
4. van Dyck CH, Swanson CJ, Aisen P, et al. Lecanemab in Early Alzheimer's Disease. N Engl J Med. 2023 Jan 5;388(1):9-21.
5. Lecanemab prescribing information Available at: https://www.accessdata.fda.gov/drugsatfda_docs/label/2023/761269s001lbl.pdf Accessed July 24, 2023.
6. Sims JR, Zimmer JA, Evans CD, et al. Donanemab in Early Symptomatic Alzheimer Disease: The TRAILBLAZER-ALZ 2 Randomized Clinical Trial. JAMA. Published online July 17, 2023. Available at : https://jamanetwork.com/journals/jama/fullarticle/2807533 Accessed July 24, 2023.

Figure S1. Representative examples of magnetic resonance imaging of A.) a case of moderate ARIA-E (clinically mild) and B.) a case of asymptomatic ICH with concurrent ARIA-E.

A. Moderate ARIA-E (mildly symptomatic)

MRI images using T2-FLAIR sequences in a participant, showing screening visit and radiologically moderate ARIA-E at week 13, which was clinically mild, evident in left temporal and occipital lobes, and resolved at week 133.

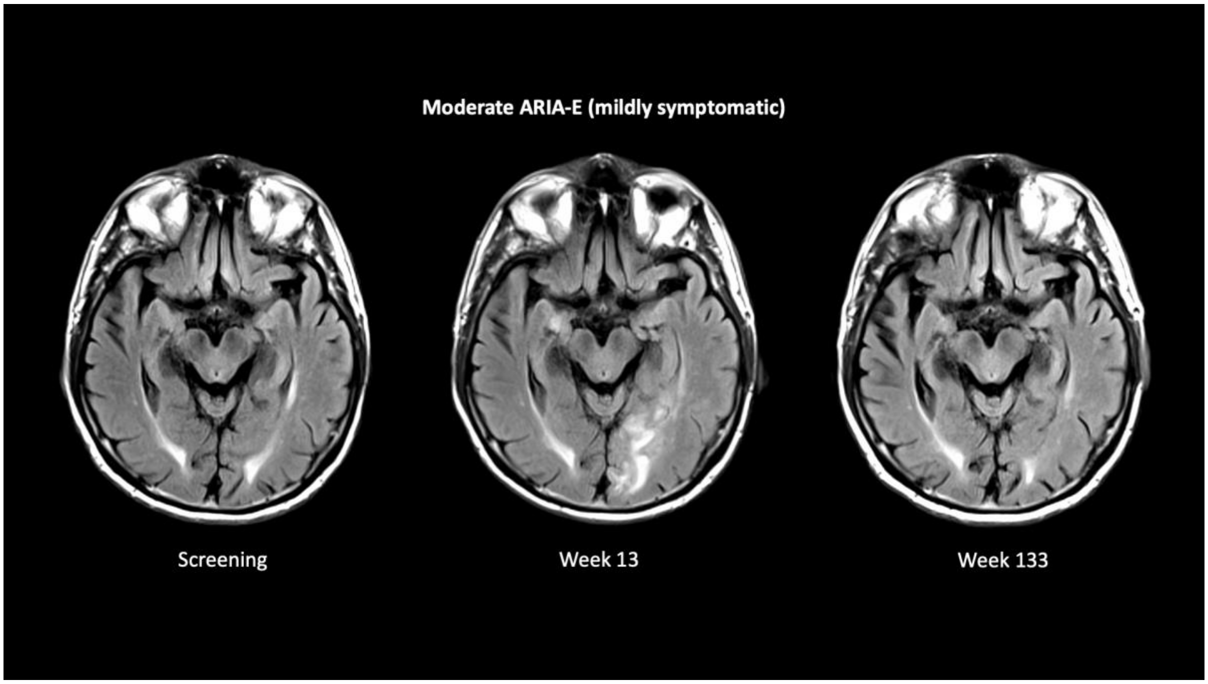

B. Asymptomatic ICH with concurrent ARIA-E (asymptomatic)

MRI images using T2-FLAIR sequences in a participant at Week 79 (left top and bottom) and Week 89 (right top and bottom). Week 89 images show development of asymptomatic ARIA-H (ICH) in the right temporal lobe (top right, see arrow), and concomitant ARIA-E sulcal effusions in the right temporoparietal, and both frontal regions (bottom right, see arrows).

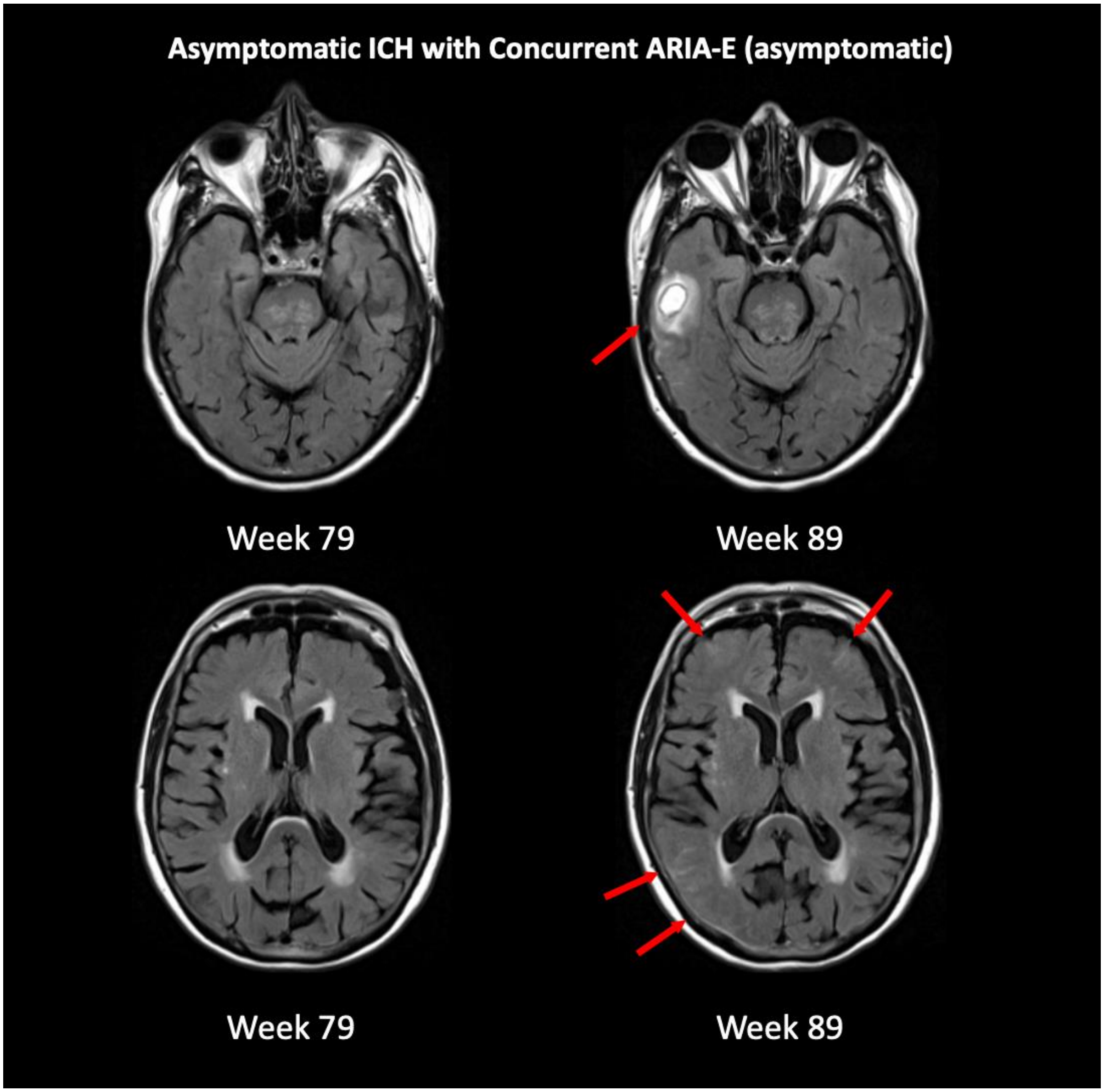

Supplement: Supplementary file 1 — Supplementary Material 1 [file 13195_2024_1441_MOESM1_ESM.pdf]
